# Spatial Variability of Geriatric Depression Risk in a High-Density City: A Data-Driven Socio-Environmental Vulnerability Mapping Approach

**DOI:** 10.3390/ijerph14090994

**Published:** 2017-08-31

**Authors:** Hung Chak Ho, Kevin Ka-Lun Lau, Ruby Yu, Dan Wang, Jean Woo, Timothy Chi Yui Kwok, Edward Ng

**Affiliations:** 1 Institute of Environment, Energy and Sustainability, The Chinese University of Hong Kong, Hong Kong, China; edwardng@cuhk.edu.hk; 2 Department of Land Surveying and Geo-Informatics, Hong Kong Polytechnic University, Hong Kong, China; 3 Institute of Future Cities, The Chinese University of Hong Kong, Hong Kong, China; 4 CUHK Jockey Club Institute of Ageing, The Chinese University of Hong Kong, Hong Kong, China; rubyyu@cuhk.edu.hk (R.Y.); 1155058927@link.cuhk.edu.hk (D.W.); jeanwoowong@cuhk.edu.hk (J.W.); tkwok@cuhk.edu.hk (T.C.Y.K.); 5 Department of Medicine & Therapeutics, The Chinese University of Hong Kong, Hong Kong, China; 6 Jockey Club Centre for Osteoporosis Care & Control, The Chinese University of Hong Kong, Hong Kong, China; 7 School of Architecture, The Chinese University of Hong Kong, Hong Kong, China

**Keywords:** geriatric depression, high-density living, socio-environmental vulnerability, urban environment, spatial analytics, urban wellbeing

## Abstract

Previous studies found a relationship between geriatric depression and social deprivation. However, most studies did not include environmental factors in the statistical models, introducing a bias to estimate geriatric depression risk because the urban environment was found to have significant associations with mental health. We developed a cross-sectional study with a binomial logistic regression to examine the geriatric depression risk of a high-density city based on five social vulnerability factors and four environmental measures. We constructed a socio-environmental vulnerability index by including the significant variables to map the geriatric depression risk in Hong Kong, a high-density city characterized by compact urban environment and high-rise buildings. Crude and adjusted odds ratios (ORs) of the variables were significantly different, indicating that both social and environmental variables should be included as confounding factors. For the comprehensive model controlled by all confounding factors, older adults who were of lower education had the highest geriatric depression risks (OR: 1.60 (1.21, 2.12)). Higher percentage of residential area and greater variation in building height within the neighborhood also contributed to geriatric depression risk in Hong Kong, while average building height had negative association with geriatric depression risk. In addition, the socio-environmental vulnerability index showed that higher scores were associated with higher geriatric depression risk at neighborhood scale. The results of mapping and cross-section model suggested that geriatric depression risk was associated with a compact living environment with low socio-economic conditions in historical urban areas in Hong Kong. In conclusion, our study found a significant difference in geriatric depression risk between unadjusted and adjusted models, suggesting the importance of including environmental factors in estimating geriatric depression risk. We also developed a framework to map geriatric depression risk across a city, which can be used for identifying neighborhoods with higher risk for public health surveillance and sustainable urban planning.

## 1. Introduction

Depression is an affective disorder commonly found among the senior population, as a result of increasing excess mortality and use of medical services [1,2,3,4,5,6]. It is also a factor that can influence other geriatric diseases, such as bone mineral density, respiratory diseases, and strokes [7,8,9]. For example, a previous study in Hong Kong indicated that approximately 8% of the senior population were affected by depression and there was a positive correlation between the prevalence of depression and the number of chronic medical conditions [10]. Among all the risk factors, social vulnerability such as older age, lower education, social isolation, and gender had the most significant influence on geriatric depression risk [6,10,11,12,13,14,15]. However, most cohort studies on the relationship between social vulnerability and geriatric depression did not consider environmental influence, which was found to affect mental health and depression by long-term deprivation of desirable life [11]. In contrast, a number of community health studies found significant associations between urban environment, vegetation cover and mental health. Better mental health was observed among people living in areas with more green spaces [16,17], and Guite et al. [18] found that dissatisfaction of access to green space and community facilities was associated with higher mental health risk. Previous studies also widely suggested that more high-rise buildings and higher residential density across a city were associated with higher level of psychological stress and psychiatric disorders [19,20,21,22]. Therefore, keys to accurately estimate geriatric depression risk are to develop a cross-sectional model incorporating both social and environmental parameters and to locate the higher-risk areas based on the estimation of socio-environmental vulnerability.

Data-driven approaches to map local areas with higher vulnerability are commonly used for preventive healthcare and emergency planning [23,24,25,26]. These vulnerability maps can be used for identifying significant hotspots of different health risks, and were previously applied to government-based protocols for the purpose of: (1) targeting areas with higher general health risk of vulnerable population [27,28,29,30,31,32,33]; (2) locating higher risk areas during specific events such as violent trauma, heat waves and extreme pollution events [34,35,36,37,38,39,40,41,42,43,44,45]; and (3) predicting spatial variability of non-communicable diseases such as cancer [46,47,48]. However, data-driven methods to predict the spatial variability of geriatric depression have rarely been investigated, leading to insufficient preventive measures to such an increasingly common disease among the senior population. Thus, it is important to develop a data-driven approach for mapping the geriatric depression risk in order to better target high-risk areas and vulnerable populations across a city.

We hereby conducted a cross-sectional analysis by combining environmental data and health outcome data from a public health survey to develop a socio-environmental vulnerability index for mapping the geriatric depression risk in Hong Kong. The specific objectives of our study are: (1) to evaluate the risk of geriatric depression from both social and environmental factors; (2) to develop a socio-environmental vulnerability index with an innovative approach, based on estimated risks of all significant social and environmental factors; and (3) to map the geriatric depression risk based on the socio-environmental vulnerability index for the planning of preventive healthcare for geriatric depression.

## 2. Data and Methods

### 2.1. Data Collection

The health outcome data were obtained from a cohort study based on a questionnaire-based survey approved by the Clinical Research Ethics Committees of the Chinese University of Hong Kong [49]. We recruited 2000 Chinese males and 2000 Chinese females in Hong Kong, aged 65 years and above, between August 2001 and March 2003 and between 2002 and 2004, respectively, to conduct this questionnaire survey. Recruitment notices in community centers for older adults and housing estates were used to recruit the subjects. The study was conducted in accordance with the Declaration of Helsinki, and the protocol was approved by the Clinical Research Ethics Committee of The Chinese University of Hong Kong (Project identification code: CRE-2003.102).

In addition, a land utilization map was obtained from the Hong Kong Planning Department, with a 10-m spatial resolution. Vegetation cover information was retrieved from a reclassified Normalized Difference Vegetation Index (NDVI) map derived from IKONOS multispectral images (15 m), using the thresholds for vegetation cover suggested by previous local studies [50,51,52]. Building information was obtained from the Hong Kong Planning Department.

### 2.2. Measures

The 15-item Geriatric Depression Scale (GDS-15) was used as a measure of depression in this study. GDS-15 is a short-form health survey developed for screening elderly subjects for depression [53,54,55]. Asian subjects scored 8 or above were commonly identified as “in need of depression detection” [54].

The following socio-economic factors were retrieved from the cohort and used in this study: (1) age; (2) gender; (3) marital status; (4) educational level; and (5) living status (living alone or living with others. They were further converted to the following social measures to represent social vulnerability associated with ages, gender, marital status, educational level, and social isolation: older ages (aged ≥ 80); male; not married (either single, devoice, widowed or separated); low education (elementary school graduate or below); and living alone.

Four environmental measures were also added into the analytic dataset: (1) percentage of residential area (pct. residential), for the use of demonstrating a compact living environment verse rural setting; (2) percentage of vegetation (pct. vegetation); (3) average building height (avg build height); and (4) variation of building height (std build height), represented by standard deviation of building height. These four measures represent the conditions of the built environment within an approximately 400-m-radius of subjects’ addresses. They were selected in this study due to the complex urban environment in Hong Kong. For example, rural areas and areas with higher living quality in Hong Kong usually are generally characterized by low-density, low-rise buildings, while historical urban areas in Hong Kong are of higher density with lower building height. These historical urban areas may also have higher variation of building heights because of the unplanned redevelopment of certain spots within the neighborhood, in contrast to the planned new town characterized by higher building height with less variation in building types. Therefore, the combination of the four measures above represents a hypothetically comprehensive urban environment of Hong Kong.

In detail, data of residential area were retrieved from the land utilization map of Hong Kong. Areas labeled as “private residential”, “public residential” and “rural settlement” on the land utilization map were identified as “residential area”, while the other areas were identified as “non-residential area”. A binary map of residential area was then created and used for calculating the percentage of residential area. Building height and footprint in shapefiles were retrieved from the building information obtained from the Hong Kong Planning Department. Such information was then converted from vector to raster format with 1-m spatial resolution for further processing. The maps of residential area, buildings, and vegetation cover were then resampled from their original spatial resolution into 1-m resolution. Based on residential location of each subject, we estimated the pct. residential, pct. vegetation, avg build height and std building height with a 400-m radius.

The following lifestyle and health confounders were also retrieved from the cohort study: (1) physical activity; (2) alcohol consumption; (3) smoking status; (4) dementia; (5) cardiovascular-related diseases; and (6) respiratory-related diseases. These were common factors related to lifestyles and medical history that may influence geriatric depression among older adults in Hong Kong, according to previous studies [8,9,10]. Physical Activity Scale for Elderly questionnaire was used to measure physical activity [9]. Cigarette and alcohol consumption was self-reported by the subjects, with the duration and amount of past and current use of tobacco and alcohol obtained from validated methods [10]. Medical history of cardiovascular- and respiratory-related diseases was also retrieved from subjects’ medical record.

### 2.3. Analytic Approach

We developed a cross-sectional model to investigate the potential influence of social and environmental vulnerability to geriatric depression risk. Odds ratios (OR) estimated by a binomial logistic regression were used to estimate the relative risk from socio-environmental vulnerability. All analyses were conducted in R software with the glm2 package [56]. In this study, we defines subjects with GDS-15 ≥ 8 as cases and the subjects with GDS-15 < 8 as controls, based on the report of Lee et al. [54] that this cutoff is more appropriate to determine geriatric depression of Asian populations. We also normalized avg build height and std build height to 100% percentiles for model estimation. In addition, all variables related to lifestyles and medical history were applied to the regression as confounding factors.

$$\begin{array}{ll} \text{log}(\frac{\text{Depression}}{\text{Not Depression}}) & \sim \;\beta_{0}+\beta_{1}\times \text{Older Ages}+\beta_{2}\times \text{Male}+\;\beta_{3}\times \text{Not Married}+\beta_{4}\\ & \times \;\mathrm{Low}\;\mathrm{Education}+\;\beta_{5}\times \mathrm{Living}\;\mathrm{alone}+\beta_{6}\times \mathrm{pct}.\;\mathrm{residential}+\;\beta_{7}\\ & \times \;\mathrm{pct}.\text{ vegetation}+\beta_{8}\times \mathrm{pct}.\text{ avg build height}+\beta_{9}\times \mathrm{pct}.\text{ std build height}\\ & +\;\beta_{10}\times \text{dementia}+\;\beta_{11}\times \text{physical activity}+\;\beta_{12}\times \text{cardiovascular diseases}\\ & +\;\beta_{13}\times \text{respiratory diseases}+\;\beta_{14}\times \text{alcohol consumption}+\;\beta_{15}\\ & \times \;\mathrm{smoking}\;\mathrm{status} \end{array}$$

ORs with 95% confidence intervals (CIs) were reported to indicate the relative risk from each type of the vulnerability. Data with missing values were excluded from this cross-sectional analysis. We compared the adjusted ORs above (Model 3) with the crude ORs of each social and environmental factors, also with the adjusted ORs of models with only social vulnerability factors (Model 1) or only environmental factors (Model 2), for the purpose of further discussion.

### 2.4. Socio-Environmental Vulnerability Index: Development and Validation

Based on the ORs from the cross-sectional analysis, we chose the variables with significantly elevated risk as the parameters to construct the socio-environmental vulnerability index. We weighted the socio-environmental vulnerability factors by their percentage increase of risk from the ORs. The spatial variation of the socio-environmental vulnerability was estimated by using the constructed index, the 2011 census data acquired from the Hong Kong Census and Statistics Department, and the environmental maps we used in this study. We also spatially overlaid the socio-environmental vulnerability map with the district map based on the finest planning unit of Hong Kong, named as “Tertiary Planning Unit” (TPU), for the purpose of further discussion.

To validate the accuracy of socio-environmental vulnerability index, we: (1) extracted the values on the produced map by the locations of health outcome data; (2) estimated the depression risk with the same cohort data by only using socio-environmental vulnerability index as the independent variable; (3) analyzed the correlation between depression risk and socio-environmental vulnerability index based on OR and CIs; and (4) reported the percentage increase of geriatric depression risk when there was 0.1 point increase in socio-environmental vulnerability index.

## 3. Results

### 3.1. Data Summary

We first used ArcGIS 10.3 to geocode data of each subject acquired from the questionnaire survey to a point-based spatial data, according to their home addresses. In total, 3944 records out of 4000 records (98.6% of the total data) were successfully geocoded. After removing data with missing values, 3930 subjects were included in the model for the estimation of depression risk (98.3%). In summary, there were 364 subjects identified as geriatric depression and 3566 subjects identified as controls in our analytic dataset (Table 1). In total, 2310 subjects resided in the historical urban areas (Kowloon and Hong Kong Island), while 1620 subjects resided in the historical rural areas (New Territories).

Comparing cases to controls, older ages, living alone, low education, not married, and pct. residential are the variables with significant difference in mean between case and control groups. In addition, there are only few significant correlations in our analytic dataset (Table 2). Among all variables, avg build height is significantly correlated with std build height (*r* = 0.82). Marital status also has a fairly positive correlation with living alone (*r* = 0.55). Considering only two pairs of variables with significant correlations, our cross-sectional model has a relatively low chance of multicollinearity.

### 3.2. Geriatric Depression Risk

In general, the crude ORs of all variables are very different from the adjusted ORs controlled by lifestyles, medical history, social vulnerability and environmental factors (Table 3). For example, our results showed that older adults, who were not married, aged older than 80 and living alone had significant geriatric depression risk based on the crude ORs but did not have significant risk based on the adjusted ORs (Models 1–3). There were also changes in the adjusted ORs for social vulnerability factors between models with (Model 1) and without (Model 2) environmental factors, which indicates that the geriatric depression risk is controlled by multiple social and environmental factors. Therefore, it is necessary to include both social and environmental factors as covariates for estimating the independent effect of each variable.

For the model controlled by medical history and lifestyle, social vulnerability and environmental factors (Model 3), older adults with higher geriatric depression risk were the subjects with lower education level, with OR of 1.60 (1.21, 2.12). Conditions of the built environment within a 400-m radius of subjects’ addresses also contributed to geriatric depression risk. Subjects who lived in an area with 1% increase in the variation of building height had 3% increase in geriatric depression risk, and subjects who lived in an area with 1% increase in percentage of residential area would have 1% increase in geriatric depression risk. In contrast, subjects living in an area with 1% increase in average building height had lower geriatric depression risk (OR: 0.98 (0.96, 0.99)). This combination of environment was mostly found in areas with extremely old buildings, namely “Tong Lau”, in Hong Kong. Tong Lau is a historical building type with no elevators, insufficient social facilities, poor building services and ventilation.

### 3.3. Socio-Environmental Vulnerability Index

Based on the results of the cross-sectional model controlled by all confounding factors, we chose the social and environmental variables that are statistically significant to develop the socio-environmental vulnerability index. Each variable was weighted based on its adjusted OR subtracted by 1. Since the OR of low education was originally estimated based on a binary variable, we further divided the weight by 100 in order to determine the change of risk due to 1% increase in low education’s population:

$$\begin{array}{ll} \mathrm{Socio}-\mathrm{environmental}\;\mathrm{vulnerability}\;\mathrm{index} & =\;0.006\times \mathrm{pct}.\;\mathrm{low}\;\mathrm{education}+0.01\times \mathrm{pct}.\;\mathrm{residential}-0.02\\ & \times \;\mathrm{pct}.\;\mathrm{avg}\;\mathrm{build}\;\mathrm{height}+0.03\times \mathrm{pct}.\;\mathrm{std}\;\mathrm{build}\;\mathrm{height} \end{array}$$

where pct. low education is the percentage of the population with low education (elementary school graduated or below) in a TPU; pct. residential is the percentage of residential area in the TPU; pct. avg build height is the normalized average building height; and pct. std build height is the normalized standard deviation of building height.

**Figure 1:**
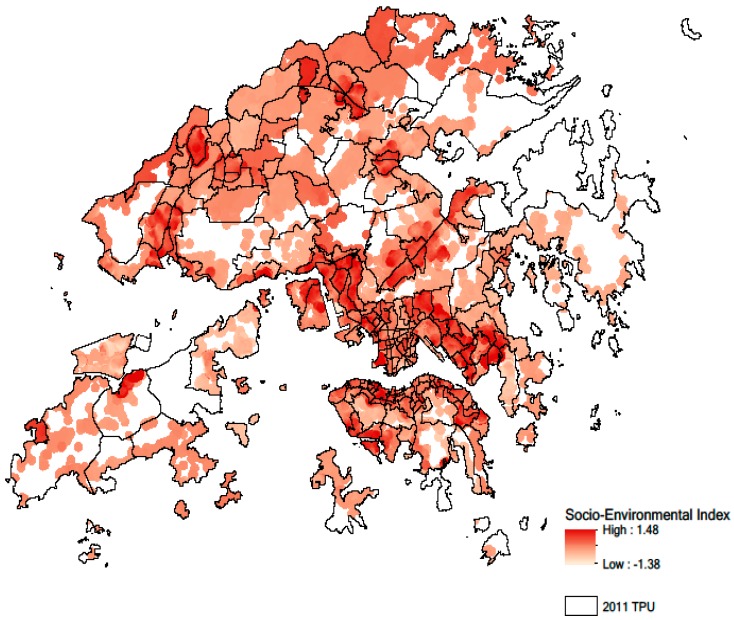
Socio-environmental vulnerability map showing the Tertiary Planning Unit (TPU) with different levels of geriatric depression risk. Note that areas without human settlements are in white color.

Based on the above index, the spatial variation of socio-environmental vulnerability was obtained using the census data and environmental data at 10-m resolution (Figure 1). The socio-environmental vulnerability index ranged from −1.38 to 1.48, with a scale unit of 0.1. We then extracted the values on the socio-environmental vulnerability map according to the location of health outcome data for the purpose of validation. OR of this validation with the geriatric depression risk was 1.53 (1.28, 1.83), indicating a significant positive correlation between socio-environmental index and geriatric depression risk. Finally, the significant CI values obtained from the validation indicate that our socio-environmental vulnerability index is accurate to be used for predicting geriatric depression risk in Hong Kong.

## 4. Discussion

### 4.1. Advantage of the Cross-Sectional Analysis

Based on 3930 subjects from the cohort study, we examined the relationship between social vulnerability and geriatric depression by using an environmentally adjusted model. We observed that the most significant risk was found in the population who had low educational status.

We reviewed previous epidemiological literature and found that our results are more conclusive than the other studies [6,11,15,57]. Previous studies found that geriatric depression risk is associated with social vulnerability [6,11,15,57,58] but none of these studies used environmental variables as controlling factors, resulting in conflicting results in some studies [6,15,57]. For example, Roberts et al. [6] found significant geriatric depression risk in population who received low education (OR: 1.62) but Forsell [57] and Østbye et al. [15] suggested that there are no significant associations between educational status and geriatric depression. Although these studies somewhat used similar epidemiological designs, the results were not comparable due to the difference in community settings and underlying physical environment. It implies that, without considering environmental factors, their results can only partially portray the actual risk of geriatric depression from social vulnerability.

In addition, biases from community influence could not be normalized, adjusted or resolved, based on their simple model design [59]. This bias was also found in other health studies when they used either environmentally adjusted or unadjusted models to correlate with different health outcomes. For example, there is a slight underestimation of high education to general health from models unadjusted by green space, compared to the adjusted model [60]. Therefore, our study can be regarded as an innovative design which comprehensively includes the morphological parameters of the built environment (building density, vegetation, building height, and urban form) in our model of geriatric depression risk.

Another strength of our study is that we determined the relationship between the built environment and geriatric depression risk, and developed a socio-environmental vulnerability index that can be used to obtain the spatial variation of geriatric depression risk. Based on the results, we found that in high-density cities, a small neighborhood with lower buildings and greater variation of building height has positive association with geriatric depression risk, instead of green space which contributes to better mental health in mid- or low-density cities. In high-density cities, the congested living environment is often perceived to have detrimental effect on mental well-being. The present study implies that building density can be balanced by proper urban design. For instance, better design of urban spaces can encourage the use of such spaces and hence provide opportunities for recovery from psychological stresses [19].

Results of the present study also provides a more advanced and reliable understanding than previous studies which most of the vulnerability indices were either conceptually developed without validation by health outcome data [35,36] or only locally calibrated without environmental adjustment [27]. Therefore, previous indices either require further proof from public health analysis [43] or may only be able to represent a local scenario that cannot be universally applied. The present study provides a methodology of developing a socio-environmental vulnerability index that can be applied in high-density cities since both social and environmental factors are considered. The conceptual framework of this study can also be used to develop similar indices for other types of urban environment.

### 4.2. Relationship between Geriatric Depression and Local Environment

The results of the cross-sectional analysis were then used to construct the socio-environmental vulnerability index. The importance of geographical scale in spatial mapping was widely discussed in previous studies [61,62]. In this study, individual responses are capable of representing the socio-economic characteristics of the neighborhood due to the large sample size. TPUs, the mapping units used in this study, are designated for small-scale planning purpose so the built environment is relatively homogeneous in the same TPU. As such, the 400-m buffer used in acquiring the information about the built environment is representative of the TPUs that the subjects reside in. Moreover, the subjects mainly live in urban areas so our mapping results can represent the geriatric depression risk at neighborhood scale in Hong Kong.

The spatial pattern of the socio-environmental vulnerability index was found to be associated with the variations in demographic and socio-economic characteristics. In addition, previous studies suggested socio-economic conditions and access to community facilities were associated with different health impacts [18,27,28,32]. We found that areas with relatively higher vulnerability are the districts with lower socio-economic status, for example, Sham Shui Po and Kwun Tong [63]. On the other hand, richer people commonly reside in urban areas with lower vulnerability such as the Peak and Kowloon Tong. Nonetheless, there were independent contributions from the built environment after adjusting for socio-economic factors. Our environmentally adjusted model can therefore take into account the effect of the built environment for better estimation of geriatric depression risk in Hong Kong.

**Figure 2:**
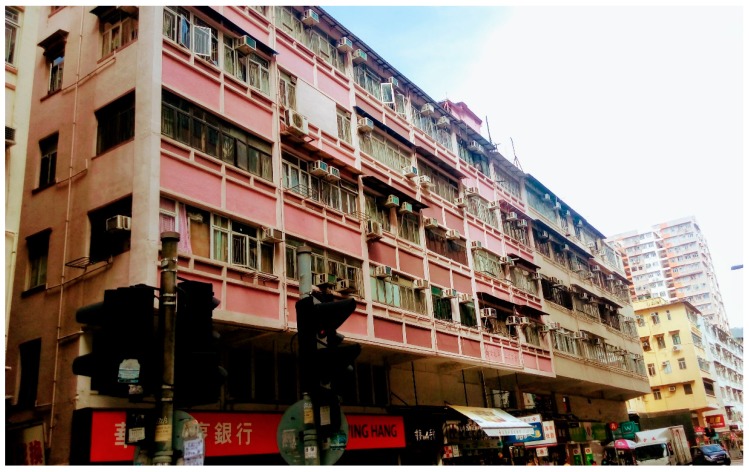
Representation of “Tong Lau”, historical buildings with poor quality and living conditions in Hong Kong. Picture captured in San Po Kong, one of the socially deprived districts in Hong Kong.

The present study indicates that there is a significant relationship between the built environment and geriatric depression risk and the density of residential buildings, average and variation of building height were identified as significantly influential factors. It highlights the characteristics of the compact living environment associated with low resilience or social vulnerability. In this study, the 25th percentile of average building height within a 400-m radius is approximate to 27.9 m which corresponds to buildings with less than ten floors. In Hong Kong, a particular type of the historical buildings called “Tong Lau” is typically with less than ten stories and located in socially deprived districts such as Sham Shui Po, San Po Kong, and Kwun Tong (Figure 2). Unlike the low-rise buildings in North America or Europe, “Tong Lau” represents a compact built environment with extremely poor living conditions, in term of building services, social environment, and living quality [64]. Some flats in these buildings could be split into only 3 to 5 m^2^ with extremely inadequate facilities. As such, it is expected that older adults living in these tiny flats would become socially isolated and suffer from depression.

**Figure 3:**
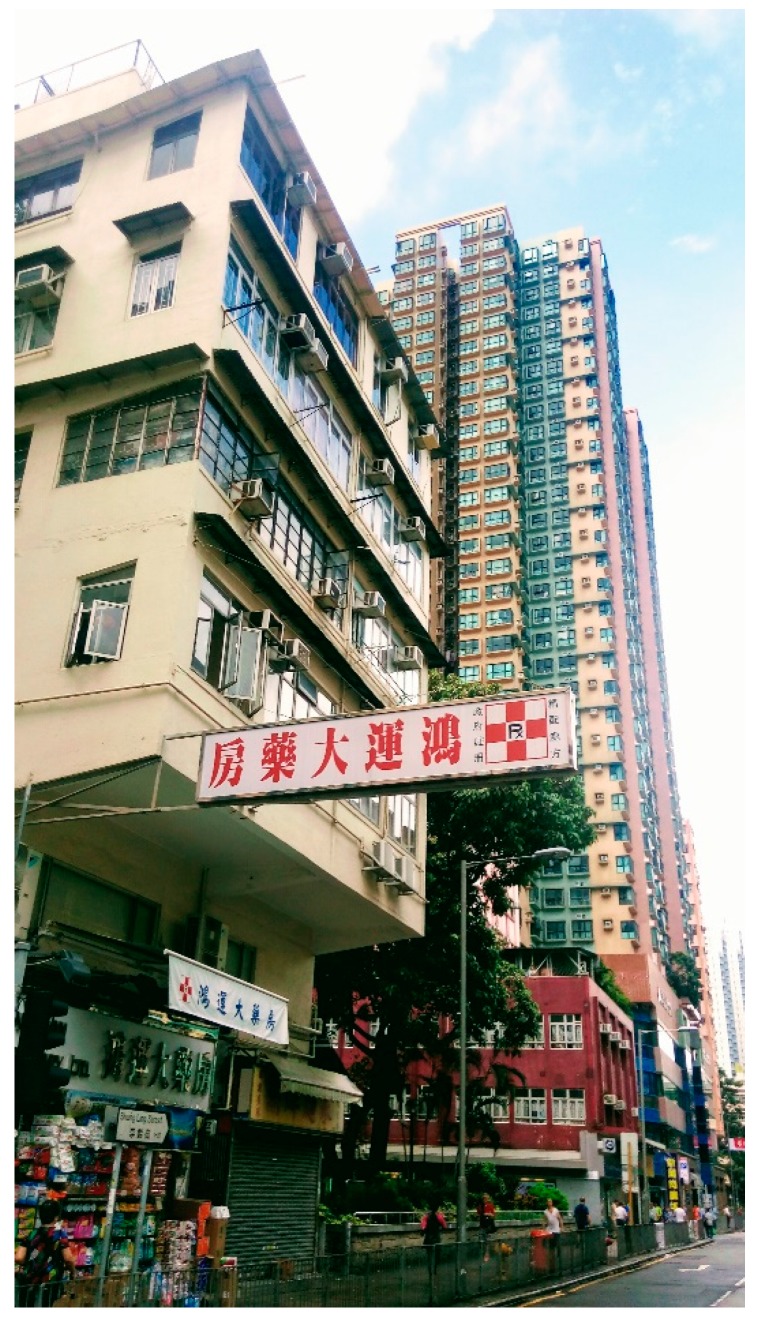
New development of single high-rise buildings in the community comprised of mostly “Long Lau”. Picture captured at San Po Kong, one of the socially deprived districts in Hong Kong.

In addition, single high-rise buildings are replacing some of the “Tong Lau” as these old urban areas experience redevelopment in recent years (Figure 3), resulting in higher variation in building height and irregular urban form. Our results indicate that high variation in building height was associated with an increased risk of geriatric depression. This can be attributed to the gentrification process that the middle class starts to occupy these areas. It accentuates social isolation due to the difference in lifestyles and changes in local communities, resulting in higher geriatric depression risk.

The variations in building height imply the current situation of redevelopment in Hong Kong. Single-building redevelopment may result in gentrification in the older urban areas and it does not provide sufficient opportunities to improve social services and facilities in the neighborhood. As for strategic planning of redevelopment in these areas, a holistic approach should be adopted to ensure that social services and facilities, even green space, are in place. It can also echo the concept of ageing in place by taking redevelopment of older districts as opportunities to improve social services and facilities in the neighborhood.

In our study, we found that there was no significant relationship between green space and geriatric depression, which is contradictory to previous studies [17,65] and likely due to the collective consideration of urban and rural areas into a single regression model. In Hong Kong, the hilly topography limits the extent of urban areas and results in very significant separation between urban and rural areas. As rural areas in Hong Kong are generally surrounded by country parks and extensive coverage of green space, combining the data of both urban and rural areas into a single model may induce biases to describe the linear relationship between green space and geriatric depression. Further studies can be conducted by using non-linear models and machine learning techniques for environmental health studies [66,67] to determine the relationship between green space and geriatric depression so that the biases of location and selection can be avoided. Nonetheless, we found that the availability of green space was coincidentally lower in more socially vulnerable areas such as Sham Shui Po, Wong Tai Sin and Kwun Tong (1.9, 1.7 and 1.3 m^2^ per each older adult, respectively), compared to 3.4 m^2^ of green space per each older adult in Hong Kong [68]. Such a high percentage of socially deprived population in these old urban areas contributes to psychological distress, which in turn increases the risk of developing depression [69].

### 4.3. Connection with Local Geriatric Studies

Consistent with previous studies in Hong Kong [70,71,72], low education was found to be associated with depressive symptoms in this study. Older adults with lower education level were likely to be depressed, indicating the importance of family relations and social support in protecting older adults from depression [70]. Therefore, strategies should be developed to facilitate social connectedness. Building social networks for older adults in vulnerable areas could be useful to prevent depression among the senior population.

Early identification and management of older adults with potential depression risk is important to reduce the risk of morbidity, disability and mortality. In Hong Kong, the prevalence of depressive symptoms/depression in the senior population is common [70,71,72,73]. However, these conditions are generally unrecognized and masked in patients with cognitive impairment or being seen as less influential. According to Lam et al. [71], only 26% of the individuals with common mental disorders in Hong Kong consulted mental health services in the past years while less than 10% of the individuals consulted general practitioners and family physicians. Mental health services in Hong Kong were especially inadequate [74], as reflected by the long waiting time (81 weeks) for a first appointment at a psychiatric out-patient clinic in 2013 [75], which may cause further geriatric depression risk to the senior population [74]. Our vulnerability map locating the areas with high geriatric depression risk estimates the levels of depression and provides useful information that can be used to guide resource allocation for mental health services.

### 4.4. Limitations

One limitation of this study is that we do not have sufficient data to analyze shifts of geriatric depression risk through time. While we normalized or adjusted the model to a setting with “same living environment”, understanding the shift will improve the flexibility of applying the socio-environmental vulnerability index. It is particularly useful for the planning of public health policy which targets long-term mitigation. The present study, nonetheless, estimates the spatial variation of the socio-environmental vulnerability under the typical scenario, which is important to preventive healthcare and emergency response [25,76]. In order to combine the advantages of both types of studies, future studies should focus on repeatedly re-collecting information of health outcomes from the participants of the cohort study and analyzing the geriatric depression risk through time based on the health outcome data collected at different times.

Another limitation of our study is that our cohort data were mostly obtained from urban areas, which induced very different results in extremely rural areas. Although our results are consistent with previous studies that the highest vulnerability in Hong Kong was found in rural areas with limited access to transportation and community facilities [18], some rural areas exhibited extremely low values based on the estimation of the socio-environmental vulnerability index. It is partially because our model only included the average and variation of building height and the results were largely determined by social variables due to the lack of building data in the rural areas. As social vulnerability highly contributes to depression risk, the spatial differences in the socio-economic structure between rural areas are relevant to the geriatric depression risk in Hong Kong.

In this study, we followed the methodology of previous studies for the development of vulnerability indices, by first examining the relationship between health risk and individual-level socio-economic status, and further applying the results with area-level data for mapping. This approach provides better ability for cohort data and health surveys to facilitate the biostatistical analyses, while it may also have issue of ecological fallacy because of the unclear relationship between individual-level data and area-level information. In this study, based on the assumption that the characteristics of the built environment in the neighborhood are homogeneous at TPU level, individual data are representative of the neighborhood conditions. Future studies can be conducted to compare the individual-level and area-level data for the representativeness to improve the development of local vulnerability indices. In addition, models for casual relationship may be an option for index improvement if comprehensive local data are available for such studies.

In addition, we did not include certain factors of environmental deprivation such as extreme temperature and air pollution in this study [77,78]. However, there are uncertainties in using fine-scale mapping of temperature and air pollution in high-density urban settings [79], which may introduce random errors to the cross-sectional model. For future studies, more information about the spatial variation of temperature can be obtained using satellite images and numerical modeling of energy fluxes in urban settings [80], while the spatial variation of air pollution can be obtained through remote sensing techniques and land use regression models [81]. These approaches provide high-quality spatial datasets that could be used for cross-sectional analyses to adjust for different factors of environmental deprivation in the future.

Finally, this study provided a framework of applying data-driven techniques to map geriatric depression risk across a city. However, variables that we used in this framework may not be applicable in other cities. For example, variation in building height is a specific variable to predict geriatric depression risk in Hong Kong due to the historical urban development of the city. This variable may not be commonly useful across other cities with similar sizes if these cities have been practicing different land use policies for regional development. Therefore, variables should be specifically chosen for the development of local vulnerability indices that can be applied to other cities.

## 5. Conclusions

We determined the relationship between the built environment and geriatric depression risk by developing a cross-sectional model consisting of five common factors of social vulnerability and four environmental factors. After minimizing bias from environmental factors, our model found that lower education was significantly associated with geriatric depression risk. Residential density, and average and variation of building height were also found to be associated with geriatric depression risk in high-density cities but green space was not a significant factor, unlike the results obtained from mid- or low-density cities. A socio-environmental vulnerability index was developed to estimate geriatric depression risk using the results of our cross-sectional model. Based on the validation of our vulnerability index, we found that an increase in the socio-environmental vulnerability index corresponds to an increase in geriatric depression risk. Further studies can be conducted to include multi-year studies to improve the flexibility of the socio-environmental vulnerability index and hence the estimation of geriatric depression risk.

## Figures and Tables

**Table 1:** Summary table for the percentage of subjects of all social variables for case and control groups. The mean and standard deviation of environmental variables based on subjects’ addresses are also presented. *p*-Values indicate if there are significant differences between case and control groups based on *t*-test.

Variables	Case (*n* = 364)	Control (*n* = 3566)	*p*-Value
Social Vulnerability			
Older Ages (Age ≥ 80)	15.4%	9.5%	<0.05
Living Alone	19.8%	12.9%	<0.05
Low Education	78.6%	65.5%	<0.05
Non-married	39.6%	28.1%	<0.05
Male	45.6%	50.5%	0.07
Environmental Vulnerability			
Pct. Residential	33.8 ± 9.7	32.5 ± 10.4	<0.05
Pct. Vegetation	24.6 ± 21.3	23.3 ± 21.2	0.28
Average Building Height (m)	33.8 ± 8.5	33.9 ± 9.1	0.84
Variation of Building Height (m)	32.1 ± 8.6	31.2 ± 9.3	0.06

**Table 2:** Correlation matrix of the analytic dataset.

Variables	OA	LA	LE	NM	Male	%Res	%Veg	ABH	SDBH
Older Ages (OA)	1								
Living Alone (LA)	0.13	1							
Low Education (LE)	0.06	0.06	1						
Not Married (NM)	0.21	0.55	0.19	1					
Male	−0.03	−0.19	−0.27	−0.38	1				
Pct. Residential (%Res)	0.03	0.08	0.07	0.06	−0.05	1			
Pct. Vegetation (%Veg)	0.01	−0.03	0.07	0.03	−0.04	−0.21	1		
Avg Build Height (ABH)	−0.02	0.06	0.03	0.07	−0.02	−0.04	0.12	1	
Std Build Height (SDBH)	−0.01	0.03	0.03	0.07	−0.03	−0.04	0.20	0.82	1

**Table 3:** Odds ratio of vulnerability variables to geriatric depression with significant variables are marked with asterisks. Models 1 and 2 are models with only social factors and only environmental factors, respectively, whereas Model 3 considers both social and environmental factors. * Indicates significant result with *p*-value < 0.05.

Variables	Crude ORs	Model 1	Model 2	Model 3
Older Ages: Age ≥ 80	1.74 (1.28, 2.36) *	1.29 (0.93, 1.80)		1.26 (0.91, 1.75)
Living Alone	1.66 (1.26, 2.19) *	1.27 (0.91, 1.78)		1.31 (0.93, 1.85)
Low Education	1.94 (1.49, 2.51) *	1.63 (1.24, 2.16) *		1.60 (1.21, 2.12) *
Not Married	1.67 (1.34, 2.09) *	1.28 (0.95, 1.72)		1.24 (0.92, 1.67)
Male	0.82 (0.66, 1.02)	1.11 (0.85, 1.46)		1.13 (0.86, 1.48)
Pct. Residential	1.01 (1.00, 1.02) *		1.01 (1.00, 1.02) *	1.01 (1.00, 1.02) *
Pct. Vegetation	1.00 (0.998, 1.01)		1.00 (0.996, 1.01)	1.00 (0.995, 1.01)
Avg Build Height	1.00 (0.99, 1.00)		0.98 (0.96, 0.99) *	0.98 (0.96, 0.99) *
Std Build Height	1.01 (0.999, 1.02)		1.03 (1.01, 1.04) *	1.03 (1.01, 1.04) *
